# Reproductive and developmental toxicities of 5-fluorouracil in model organisms and humans

**DOI:** 10.1017/erm.2022.3

**Published:** 2022-01-31

**Authors:** Gerile Naren, Jiaojiao Guo, Qiujuan Bai, Na Fan, Buhe Nashun

**Affiliations:** State Key Laboratory of Reproductive Regulation and Breeding of Grassland Livestock, School of Life Sciences, Inner Mongolia University, Hohhot, China

**Keywords:** 5-Fluorouracil, embryos, ovary, reproductive toxicity, sperm, testis

## Abstract

Chemotherapy, as an important clinical treatment, has greatly enhanced survival in cancer patients, but the side effects and long-term sequelae bother both patients and clinicians. 5-Fluorouracil (5-FU) has been widely used as a chemotherapeutic agent in the clinical treatment of various cancers, but several studies showed its adverse effects on reproduction. Reproductive toxicity of 5-FU often associates with developmental block, malformation and ovarian damage in the females. In males, 5-FU administration alters the morphology of sexual organs, the levels of reproductive endocrine hormones and the progression of spermatogenesis, ultimately reducing sperm numbers. Mechanistically, 5-FU exerts its effect through incorporating the active metabolites into nucleic acids directly, or inhibiting thymidylate synthase to disrupt the function of DNA and RNA, leading to profound effects on cellular metabolism and viability. However, some studies suggested that the toxicity of 5-FU on reproduction is reversible and certain drugs used in combination with 5-FU during chemotherapy could protect reproductive systems from 5-FU damage both in females and males. Herein, we summarise the recent findings and discuss underlying mechanisms of the 5-FU-induced reproductive toxicity, providing a reference for future research and clinical treatments.

## Introduction

Chemotherapy is a form of drug therapy meant to kill fast-growing tumour cells by powerful chemicals in the body. 5-Fluorouracil (5-FU) is one of the most commonly used chemotherapy drugs during clinical treatment of cancers in gastrointestinal tract, pancreas, ovary, oesophageal, colorectal and breast since 1957 (Refs 1–7). However, it has various side effects, such as long-term memory impairments, myelosuppression and cardiotoxicity (Refs 8–13). With the continuous development of modern oncology and pharmacology, a series of 5-FU derivatives and analogues, including 1-hexylcarbamoyl-5-fluorouracil, tegafur, uracil tegafur, capecitabine, TAS-102 (trifluridine-tipiracil) and S-1 (tegafur, gimeracil and oteracil potassium) have been developed, which could increase and maintain higher 5-FU concentration in the serum (Refs 14–18).

It is currently believed that 5-FU and its derivatives exert their anti-tumour effect mainly through inhibiting thymidylate synthase (TS) and incorporating its metabolites into DNA and RNA (Ref. 19). After administration, most of the 5-FU is metabolised into inactive dihydrofluorouracil by the rate-limiting enzyme dihydropyrimidine dehydrogenase (DPD) in the liver (Ref. 20). The rest of the administered 5-FU is transformed mainly into active metabolites of fluorodeoxyuridine monophosphate (FdUMP), fluorodeoxyuridine triphosphate (FdUTP) and fluorouridine triphosphate (FUTP). FdUMP binds with TS and the methyl donor, folate 5,10-methylenetetrahydrofolate to inhibit the normal function of TS. This ternary complex blocks deoxyuridine monophosphate binding to TS and inhibits synthesis of deoxythymidine monophosphate (dTMP). Since dTMP is a key substrate for the production of deoxythymidine triphosphate (dTTP), depletion of dTMP results in the subsequent depletion of dTTP, and induces perturbations in the levels of other deoxynucleotides (dATP, dGTP and dCTP), disrupting DNA synthesis and repair. Meanwhile, inhibition of TS also results in an increased level of dUTP, which along with the FdUTP can be misincorporated into DNA, leading to breaking of DNA strands (Ref. 21). Additionally, the 5-FU metabolite FUTP is also incorporated extensively into RNA, disrupting normal RNA processing and function (Ref. 22). Therefore, 5-FU severely disrupts synthesis, repair and function of nucleic acids through its active metabolites, inhibiting growth of cells and killing tumour cells ultimately. However, 5-FU also impairs cellular metabolism and viability in normal cells, which underlies the developmental and reproductive toxicities (Refs 23–25).

To date, several attempts have been made to alleviate the reproductive side effects of 5-FU. Apart from the combined administration of 5-FU with other drugs such as triptorelin, 6-alkylguanine-DNA alkyltransferase (AGT) and iridoids-rich containing fraction of *Pentas lanceolata* leaves (IFPL) (Refs 26–28), manipulation of gene expression including down-regulation of the uracil-DNA glycosylase (UNG) or overexpression of DPD and TS homologues also alleviates the reproductive toxicity (Refs 25, 29). Moreover, targeted delivery and controlled release of 5-FU by biocompatible and biodegradable particles represents a promising direction to overcome the side effects of 5-FU (Ref. 30). 5-FU has a narrow therapeutic window, and for this reason, selection of the appropriate dosage is crucial to reduce its side effects. In this context, the test of single-nucleotide polymorphisms (SNPs) shows great potential in optimising 5-FU dosage. In patients with *DPYD* variants (DPD coding genes) of c.190511G > A (rs3918290), c.1679T > G (rs55886062), c.2846A > T (rs67376798) and c.1129-5923C > G (rs75017182), lower dosage of 5-FU inhibits cancer cell growth and achieve equal anti-tumour efficacy (Ref. 31). Similarly, SNPs in 5-FU metabolic genes such as *CDA*, *CES2*, *TYMS* (*TS*) and *MTHFR* can also be used to predict the efficacy and toxicity of capecitabine-based therapy (Ref. 32). Therefore, genetic SNPs provide pharmacogenomics information for clinical application of 5-FU to adjust dosage, improve efficacy and reduce side effects, ultimately. However, it should be noted that though increasing efforts to alleviate reproductive toxicity of 5-FU, currently, there is no effective way to avoid the damage completely.

## Adverse effects of 5-FU on female reproduction

In *Caenorhabditis elegans*, 5-FU induces germ cell death and inhibits embryonic and larval development (Ref. 33), which presumably because of cell-cycle arrest and apoptosis of germline cells (Ref. 25). Particularly, 5-FU down-regulates expression of several collagen genes, which are important players in extracellular matrix (ECM)–receptor interaction and focal adhesion. Therefore, impairment of the ECM–receptor interaction and focal adhesion during germ cell development may underlie the 5-FU-induced fertility decline in *C. elegans* (Ref. 34). 5-FU also down-regulates LIN-29, which is an important transcription factor that affects vulva development and egg laying system (Ref. 25). Interestingly, down-regulation of UNG could alleviate the 5-FU effects on embryo hatching (Ref. 25), suggesting that UNG-mediated removal of the misincorporated 5-FU is involved in this process. Indeed, it was proposed that UNG-1 excise uracil, but the subsequent repair synthesis results in uracil reincorporation, leading to futile cycling of the base excision repair pathway (Ref. 35). Overexpression of the homologues of DPD (DPYD-1) and TS (Y110A7A.4) in *C. elegans* prevented the death of germ cells following 5-FU exposure. In contrast, depletion of DPYD-1 increased sensitivity of 5-FU and depletion of the 110A7A.4 resulted in severe embryonic lethality (Ref. 29) (Table 1). Therefore, down-regulation or overexpression of certain 5-FU metabolic enzymes could be an alternative way to reduce the reproductive toxicity.

**Table 1.** Adverse effects of 5-FU on female reproduction

Model	Dosage and treatment time	Main findings with respect to 5-FU	Sites of damage	Reference
*C. elegans*	2, 5 and 400 nM 5-FU	Overexpression of DPD and TS homologues suppressed germ cell death; DPYD-1 and Y110A7A.4 depletion resulted in embryonic death	Germ cells; embryos	Ref. 80
*C. elegans*	400 μM 5-FU soaked for 6 h; 400 nM 5-FU plated for 72 h	Germ cell death; embryonic development blocked; larvae arrest	Embryos; larvae; germ cells	Ref. 33
*C. elegans*	0.15–4.8 g/ml 5-FU	Cell-cycle arrest; germ cell apoptosis; number of mitotic nuclei per gonad arm reduced; LIN-29 expression reduced; vulva development affected; egg laying delayed	Germ cells; vulva	Ref. 25
*C. elegans*	200 mM 5-FU; 5 μM, 10 μM and 20 μM 5-FU	The embryos decreased in number and defective in development; altered gene expressions	Embryos	Ref. 34
Mice; embryos	10–40 mg/kg 5-FU	Foetal malformations; tail defects; skull defects; limb defects	Foetus; tail; limb; skull	Ref. 41
Mice; embryos	1–8 μm/ml 5-FU	Malformation frequency increased; tail dysmorphogenesis and telencephalic hypoplasia	Tail; limb; palate	Ref. 40
Mice	200 mg/kg of 5-FU	Fertility lost; lower weight pups	Fertility; pups	Ref. 36
Mice; ovary	150 mg/kg 5-FU	Secondary follicles and antral follicles increased; corpora lutea reduced; atresia rates returned to normal in 7 days	Ovary; follicle	Ref. 48
Mice; ovary	125 mg/kg 5-FU for 3 times	Growing follicles reduced; ovarian volume decreased; luteum counts reduced	Ovary; follicle	Ref. 49
Mice; ovary	450 mg/kg 5-FU in vivo; 9.2, 46.1, 92.2 mM of 5-FU in vitro	Follicles number reduced and secondary follicles lost; increased level of Bax/Bcl2, Wnt2, Wnt4; *β*-catenin immunolabelling in preantral follicles decreased	Ovary; follicle	Ref. 47
Mice; ovary	50 mg/kg FU for 4 days in vivo; 50, 100 and 500 μM 5-FU in vitro	Small ovarian size; lower number of corpus luteum; ovulation failure; oocytes maturation inhibited; embryos development reduced; all defects recovered after 1 week	Ovary; oocytes; embryos	Ref. 50
Pregnant rats	250 μg 5-FU	Foetal malformations; foetal size and weight reduction	Foetus	Ref. 42
Gestation rats; embryos	10–30 mg/kg 5-FU in vivo; 0.15–0.30 μg/ml 5-FU in vitro	Foetal malformations; hypoplastic optic vesicles	Foetus	Ref. 39
Gestation rats	0–40 mg/kg 5-FU	TS inhibition; defects in DNA synthesis; cell cycle arrest; hindlimb defects	Rats	Ref. 23
Rats; ovary	80 mg/kg 5-FU; 0.1 mg/kg triptorelin	Decreased level of AMH, Bcl-2, NF-κB, body and ovarian weight; increased level of E2, FSH and Bax	Ovary; hormones	Ref. 27
Pregnant women	5-FU inadvertent exposure	Non-toxic	–	Ref. 51
Pregnant women	2-h concurrent infusion of 85 mg/m^2^ oxaliplatin and 400 mg/m^2^ leucovorin, followed by 400 mg/m^2^ 5-FU bolus administration and 2400 mg/m^2^ 5-FU infusion	No harm to foetal health	–	Ref. 52

The mammalian fertility cycle is responsible for the coordination of various cellular events, including DNA synthesis in ovarian follicle cells, and of potential importance to the toxicity of 5-FU (Refs 36, 37). Female mice received 5-FU during the oestrous phase were suffered from greater fertility loss compared with those exposed to 5-FU during the metestrus, diestrus and proestrus stages, probably because ovarian follicular DNA synthesis is most active within the oestrous phase and 5-FU leads to pronounced DNA damage accumulation (Refs 36, 38). Therefore, it is likely that choosing an appropriate oestrus cycle for 5-FU treatment is helpful to reduce reproductive damage.

Teratogenesis is another severe reproductive abnormality induced by 5-FU. The number of externally malformed foetuses increases in a dose-dependent fashion after single administration of 10–30 mg/kg 5-FU during pregnancy in rats (Ref. 39). Studies using in vitro whole embryo culture systems also demonstrated that 5-FU dose-dependently induces tail and hindlimb bud defects, and leads to hypoplastic optic vesicles in rat embryos (Refs 39, 40). Similarly, 5-FU exposure reduces embryo implantation rate and increases embryo deformity and mortality in a dose- and time-dependent manner in mice (Ref. 41). Of note, when injected into pregnant mice, 5-FU is incorporated into the embryos and accumulates mostly in RNAs. Although varied among different strains, the incorporation amount of 5-FU is positively correlated with the weight of the embryos (Ref. 42). These findings collectively indicates that negative effects of 5-FU on embryonic development is closely related to its dosage, which in turn suggests that it is crucial to apply an optimal dosage that has expected anticancer activity but does not induce significant developmental defects (Refs 43, 44). In this context, the mouse embryonic stem cell test seemed extremely useful to assess the developmental toxicities and determine the optimal dosage of 5-FU (Ref. 45).

Cytotoxicity of 5-FU potentially contributes to the ovarian dysfunction and puts the patients at risk of menopause-related complications and infertility (Ref. 46). When young C57BL/6J female mice were injected with 5-FU, secondary follicles were lost totally (Ref. 47). Furthermore, genes involved in apoptosis and Wnt signalling pathways were significantly up-regulated when ovaries from young mice were cultured in vitro with 5-FU (Ref. 47). In adult mice, administration of 5-FU-induced atresia of secondary and antral follicles, and profoundly reduced corpus luteum counts, leading to a decreased ovarian volume (Refs 48, 49). However, primordial or primary follicles were not affected by the 5-FU treatment (Refs 48, 49), suggesting that the reproductive toxicity of 5-FU could be recovered with continuous growth of the follicles. In support of this view, we recently reported that multiple intraperitoneal administration of 5-FU in adult female mice resulted in small ovarian size and reduced number of corpus luteum, and led to ovulation failure. However, these defects could be recovered and no obvious abnormality was observed in their offspring, suggesting that the adverse effects could be reversed following withdrawal of 5-FU administration (Ref. 50). In addition to self-recovery, combined administration of triptorelin, a GnRH agonist often used as a hormone responsive anti-cancer drug, alleviates 5-FU-induced follicle number reduction, probably via decreasing the levels of E2, follicle-stimulating hormone (FSH), Bax and nuclear factor (NF)-κB, and increasing the levels of anti-Müllerian hormone (AMH) and Bcl-2 in the serum (Ref. 26). Additionally, when 5-FU was loaded in poly-glucono-*δ*-lactone particle and delivered precisely to the target sites and released in a controlled manner, the toxic effect on non-cancer cells was effectively avoided (Ref. 30).

In human clinical cases, inadvertent exposure to 5-FU (Ref. 51) or treatment with FOLFOX, a mixture of 5-FU, leucovorin and oxaliplatin, during the second and third trimesters of pregnancy had no harm to foetal health (Ref. 52). However, the developmental and reproductive toxicities of 5-FU cannot be evaluated comprehensively in humans and are assessed instead in human-induced pluripotent stem cells (hiPSCs), which has been suggested to achieve similar findings (Ref. 53). In hiPSCs, 5-FU inhibited neural differentiation via down-regulating expression of the mitochondrial fusion proteins Mfn1/2 and decreasing intracellular ATP levels (Ref. 54), suggesting that 5-FU-induced mitochondrial dysfunction may underlie the developmental and reproductive toxicities.

Adverse effects of 5-FU on the reproduction and development were also reported in amphibian, arthropods and aquatic species. Exposure to 5-FU at environmentally relevant concentrations during the early developmental stage did not adversely affect the survival or behaviour in larval zebra fish, but larvae growth represented by body length was significantly increased when exposed to higher concentration of 5-FU (Ref. 55). In *Xenopus laevis* embryos, malformations in abdominal oedema, axial flexure, head, eyes, gut and heart were observed after 5-FU treatment (Ref. 56). Moreover, 5-FU treatment resulted in reduction in the number of offspring and DNA damage in *Ceriodaphnia dubia* (Ref. 57).

In general, 5-FU inhibits embryonic or larval development in *C. elegans*, arthropods, amphibian, mouse and rat, and dose-dependently induces embryonic malformation in mice and rat. Of note, 5-FU impairs ovarian function and leads to ovulation failure, but the negative effects could be reduced by combinatorial drug administration or eliminated naturally after 5-FU withdrawal in mice. However, the underlying mechanisms remain largely unknown and the potential long-term effect could not be ruled out.

## Side effects of 5-FU on male reproduction

Usually, chemotherapy is toxic for testicular tissue and increases the risk of infertility in males (Ref. 58). Studies in mice demonstrated that 5-FU induces morphological changes of Sertoli cells and reduces weights of reproductive organs including seminal vesicle and prostate. This effect is probably mediated by hormonal imbalance in the serum, whereby the levels of GnRH and pro-alpha C were remarkably increased, whereas the levels of testosterone, activin A, prolactin and inhibin B were significantly decreased (Table 2) (Ref. 59). 6-Mercaptopurine (6-MP) is an antimetabolite drug as the 5-FU, which induces ROS, activates caspase 3 and promotes apoptosome generation, ultimately leading to the loss of Leydig cells in mice (Ref. 60). Therefore, it is tempting to speculate that whether 5-FU also impairs the Leydig cells that produce testosterone through similar mechanism.

**Table 2.** Adverse effects of 5-FU on male reproduction

Model	Dosage and treatment time	Main findings with respect to 5-FU	Sites of damage	Reference
Mice	25 mg/kg B.4152 (CNU + 5-FU)	Spermatocytes and spermatids depletion	Spermatocyte; spermatid	Ref. 27
Mice	75 mg/kg 5-FU + 100, 200 and 300 mg/kg IFPL	Percentage of CAs and morphological sperm defects increased; IFPL alleviated the defects	Sperms	Ref. 28
Rats	130 mg/kg 5-FU	Spermatid development arrest; abnormally shaped spermatids; sperm release failure; increased Sertoli cell lipid; malorientation of spermatids	Testis	Ref. 63
Rats	10, 50 and 100 mg/kg 5-FU	Epithelium sloughing; giant cell formation	Epithelium	Ref. 61
Rats	10, 50 and 100 mg/kg 5-FU	Testes weight, STD and SEH decreased; atrophic tubules; germ cells exfoliated	Testis; tubule; germ cells	Ref. 62
Rats	0, 20 and 30 mg/kg 5-FU for 2-week or 4-week	Degeneration of seminiferous epithelium; weight of the seminal vesicle and prostate reduced; testosterone level decreased; activin A and prolactin decreased; GnRH and pro-alpha C increased; Serum inhibin B decreased	Testis plasma hormones	Ref. 59
Rats	10, 20 and 30 mg/kg 5-FU	Sperm count decreased	Epididymis	Ref. 64

In male rats, 5-FU induces sloughing of epithelium and promotes giant cell formation (Ref. 61), accompanied by a significant decrease of the testis weights (Ref. 62). Moreover, tubular shrinkage, atrophy and abnormal sperm cells were also observed following 5-FU administration (Refs 62, 63), eventually leading to a significant reduction of spermatocytes/spermatids cell count in a dose- and time-dependent manner (Ref. 64). Although direct mechanistic evidence is missing for 5-FU-induced reproductive toxicity in males, 6-MP has been reported to induce DNA damage in rat spermatocytes (Ref. 65), suggesting that 5-FU may potentially induce DNA damage in spermatocytes. Moreover, 5-FU-induced swelling and crazing of tubules (Ref. 66) is reminiscent of the alkylating agent cisplatin-induced degenerative changes in seminiferous tubules and germ cell depletion, mediated by aggravated oxidative damage (Refs 66, 67). Therefore, it will be interesting to test whether 5-FU-induced abnormal seminiferous tubules is also because of the free radical-associated oxidative stress.

Importantly, combinatory use of certain agents also relieves the side effects of 5-FU on male reproduction. *N*-2-Chloroethyl-*N*-nitrosourea (CNU) is an alkylating agent often used in combination with 5-FU against a range of cancers (Ref. 68). The B.4152, composed of 5-FU and CNU, only induces minor damage in spermatogenic tissue in mice when administrated with AGT. AGT is a DNA repair protein that repairs mutagenic lesions in DNA and protects testis from alkylating agent-induced damage (Ref. 27). Another agent IFPL, which is the iridoids-rich fraction of *P. lanceolata* leaves, also plays protective role during 5-FU-induced sperm defects (Ref. 28). Furthermore, several extracts from medicinal plants and herbs possess antioxidant, anti-inflammatory or anti-oedematous activities, and potentially protect sperm from inflammation and oxidative stress induced by chemicals such as 5-FU, and reduce the adverse effects (Refs 69, 70).

The report regarding the effects of 5-FU on human male reproduction is extremely limited, but some other chemotherapy agents have been reported to exert non-negligible adverse effects. Cisplatin treatment resulted in a remarkable reduction of the number of germ cells both in human foetal and prepubertal testis, which involves an initial loss of gonocytes followed by a significant reduction in spermatogonia (Ref. 71). Paclitaxel, a taxane-based chemotherapy drug, reduced serum inhibin B and testicular volume, while elevated serum FSH level in male patients (Ref. 72). Therefore, it is reasonable to speculate that 5-FU, as a chemotherapeutic agent, also negatively affects male reproduction. Given the current status that lacking mechanistic studies of the 5-FU-induced reproductive toxicities in males, the integrated multi-organoid body-on-a-chip system containing male reproductive organoid would be a desirable model to systematically investigate the potential mechanisms of 5-FU toxicity on male reproduction (Refs 73, 74).

## Conclusion and perspectives

Collectively, 5-FU causes reproductive and developmental toxicities mainly via disrupting cellular functions and inducing hormonal imbalance. The adverse effects could be alleviated by combinatory administration of certain agents or eliminated naturally following 5-FU withdrawal, probably because 5-FU has minor gonadal toxicity compared with other chemotherapy agents and induces a lower degree of gonadal damage (Ref. 75). However, long-term reproductive effect of 5-FU treatment is still under debates and further in-depth evaluation including systematic analysis of health condition and life span of the descendants should be performed. Given the superior advantages of nanocarriers in drug delivery and release, it will be crucial to develop novel, efficient nanocarriers to reduce the reproductive and developmental toxicities of 5-FU (Refs 76–79). Moreover, metabolic and multiomics analysis combined with organoid studies will be a promising strategy to elucidate mechanistic details of the 5-FU-induced reproductive toxicities.
